# Genome-wide expression assay comparison across frozen and fixed postmortem brain tissue samples

**DOI:** 10.1186/1471-2164-12-449

**Published:** 2011-09-10

**Authors:** Maggie L Chow, Hai-Ri Li, Mary E Winn, Craig April, Cynthia C Barnes, Anthony Wynshaw-Boris, Jian-Bing Fan, Xiang-Dong Fu, Eric Courchesne, Nicholas J Schork

**Affiliations:** 1 Department of Neuroscience, NIH-UCSD Autism Center of Excellence, School of Medicine, University of California San Diego, 8110 La Jolla Shores Dr Ste 201, La Jolla, CA 92093, USA; 2 Department of Cellular and Molecular Medicine, School of Medicine, University of California San Diego, 9500 Gilman Drive # 0651, La Jolla, CA 92093, USA; 3 Scripps Genomic Medicine & The Scripps Translational Sciences Institute (STSI), The Scripps Research Institute, 3344 North Torrey Pines Court, Room 306, La Jolla, CA 92037, USA; 4 Graduate Program in Biomedical Sciences, Department of Medicine, University of California at San Diego, La Jolla, CA 92093, USA; 5 Illumina, Inc. 9885 Towne Centre Drive, San Diego, CA 92121, USA; 6 Division of Medical Genetics, Department of Pediatrics and Institute of Human Genetics, University of California San Francisco, School of Medicine, Box 0794, Core Campus, HSE 901F, San Francisco, CA 94143- 0794, USA

**Keywords:** Brain, Gene Expression, DASL, IVT

## Abstract

**Background:**

Gene expression assays have been shown to yield high quality genome-wide data from partially degraded RNA samples. However, these methods have not yet been applied to postmortem human brain tissue, despite their potential to overcome poor RNA quality and other technical limitations inherent in many assays. We compared cDNA-mediated annealing, selection, and ligation (DASL)- and *in vitro *transcription (IVT)-based genome-wide expression profiling assays on RNA samples from artificially degraded reference pools, frozen brain tissue, and formalin-fixed brain tissue.

**Results:**

The DASL-based platform produced expression results of greater reliability than the IVT-based platform in artificially degraded reference brain RNA and RNA from frozen tissue-based samples. Although data associated with a small sample of formalin-fixed RNA samples were poor when obtained from both assays, the DASL-based platform exhibited greater reliability in a subset of probes and samples.

**Conclusions:**

Our results suggest that the DASL-based gene expression-profiling platform may confer some advantages on mRNA assays of the brain over traditional IVT-based methods. We ultimately consider the implications of these results on investigations of neuropsychiatric disorders.

## Background

Gene expression profiling investigations involving postmortem brain tissue of cases with neuropsychiatric disorders such as autism have been limited due to tissue availability and tissue quality [1-3]. Such investigations, however, are critical for understanding uniquely human disorders [4]. While experimenters cannot control tissue availability, novel technologies can be employed to utilize the precious and scarce tissue resources available from brain banks even if preservation quality is not ideal [5-7].

The cDNA-mediated annealing, selection, and ligation (DASL) gene expression assay has been shown to produce highly reliable results when applied to formalin-fixed, paraffin-embedded tissues [7,8]. To overcome the difficulties associated with poly A/oligo-dT-based priming in special experimental conditions such as with profiling partially degraded RNA, the DASL-based assay uses random priming at the cDNA synthesis step. It generates first strand cDNA to minimize variation during random priming and avoids biases associated with sample amplification and labeling from multiple rounds of random priming [9]. Furthermore, the assay requires only a ~50 nucleotide target sequence for query oligonucleotide annealing, which makes it effective for quantifying partially degraded RNA samples.

The DASL-based methodology has already been applied in the study of human liver, esophagus, breast, prostate, ovarian, and other biopsy and autopsy tissues [8,10-13]. It can be extended for use in a genome-wide format, which may be of value in the elucidation of genes mediating neuropsychiatric diseases [4]. However, it has not yet been applied to postmortem frozen and formalin-fixed brain tissue, despite its potential benefits when assaying samples with low RNA quality.

In this study, we investigated the utility of the standard IVT- and DASL- based genome-wide expression profiling assays in the context of a clinically important neuropsychiatric disorder, autism. Our objectives were to: 1) compare the quality of microarray data from IVT-based and DASL-based platforms on artificially degraded reference RNA; and 2) compare the quality of microarray data from these two RNA profiling platforms on postmortem frozen and formalin-fixed brain tissue.

## Results

### DASL-based expression profiling is more reliable than IVT-based profiling on artificially degraded brain reference RNA

To first assess the reliability of IVT- and DASL-based platforms in expression profiling of artificially degraded reference RNA samples, we performed these two assays on brain and pooled reference RNA heated at 95°C for 0 (intact RNA), 10, 30, and 60 minutes (Additional File 1). Heating fragments the reference RNA, which simulates RNA degradation conditions *in vivo*. The more time that RNA is subjected to heating, the more fragmented the RNA becomes (Additional File 1).

**Figure 1:**
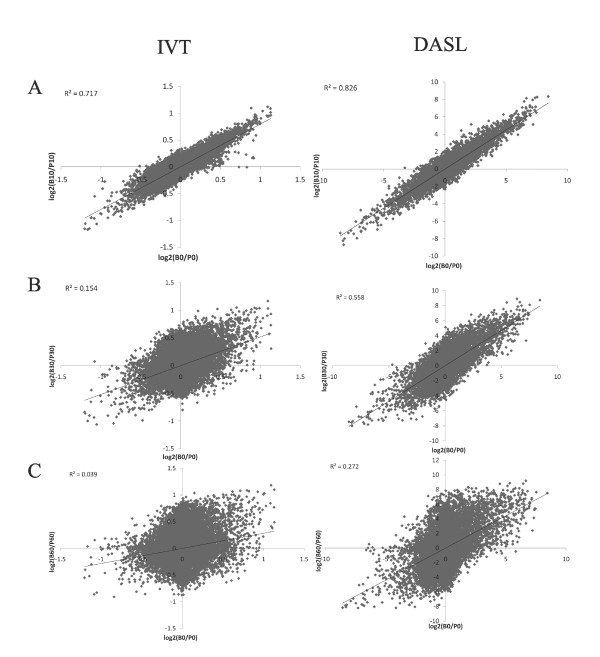
**Fold change differences between degraded brain and pooled reference RNA samples show greater similarity to intact RNA in profiling by DASL- (right column) than by IVT-based (left column) approach**. Scatterplots depicting unnormalized log2 fold change [Log2(Brain/Pooled)] of detected intensity values between intact brain and pooled reference RNA samples, and correlations (R^2^) with artificially degraded brain and pooled reference RNA samples at 10 min (A), 30 min (B) and 60 min (C) are shown. For example, the log fold change difference between brain and pooled RNA heated for 60 minutes was correlated with the intact RNA R^2 ^= 0.039 using the IVT-based approach, but R^2 ^= 0.272 using the DASL-based approach. B, brain reference RNA; P, pooled reference RNA; 0, intact; 10, heated at 95°C for 10 min; 30, heated at 95°C for 30 min; 60, heated at 95°C for 60 min.

Fold change differences between brain reference RNA and pooled reference RNA were used to assess the reliability of gene expression profiles at different levels of RNA fragmentation (Figure 1). The IVT-based assay yielded low correlations between expression profiles detected in intact RNA, which was used as the standard, and degraded RNA. Correlation with intact RNA yielded a correlation coefficient R^2 ^= 0.717 at 10 minutes of heating, R^2 ^= 0.154 at 30 minutes, and R^2 ^= 0.039 at 60 minutes. In contrast, even extremely degraded RNA profiled by the DASL-based assay yielded higher correlations with intact RNA. Correlation with intact RNA yielded a correlation coefficient R^2 ^= 0.826 at 10 minutes of heating, R^2 ^= 0.558 at 30 minutes, and R^2 ^= 0.272 at 60 minutes.

**Figure 2:**
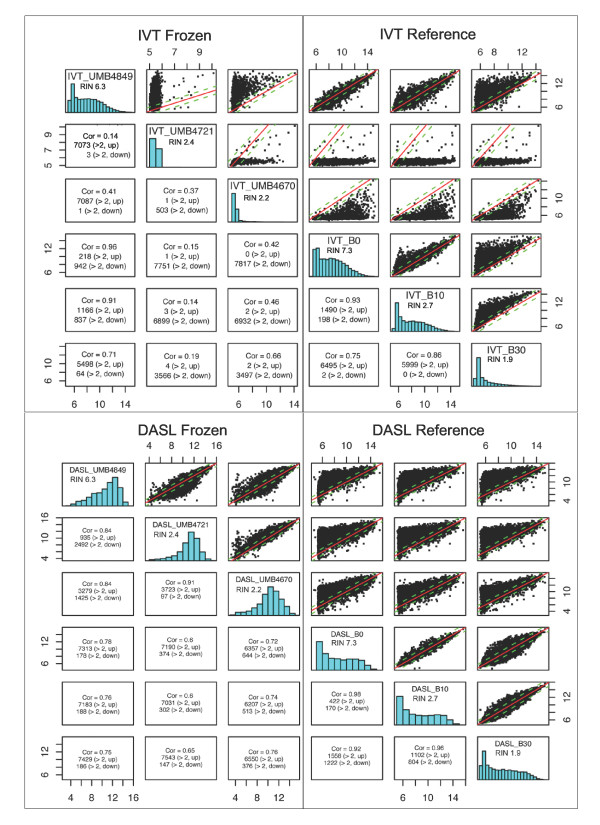
**Assay performance on RNA from frozen tissues and artificially degraded reference RNA**. DASL-based expression profiling produces higher correlations between frozen tissue-based RNA samples and reference RNA samples regardless of RNA degradation. Scatterplots, histograms, and correlation of frozen tissue-based RNA and reference RNA profiling on IVT- and DASL-based platforms are shown. For example, correlation between UMB4849 (RIN = 6.3) and UMB4721 (RIN = 2.4) is 0.14 for IVT (four top left boxes) but 0.84 for DASL (four top left boxes under "DASL Frozen"). For the IVT-based samples, histogram shows clustering of detected intensities for UMB4721 at background levels, and skewing of the scatterplot away from y = x. In contrast, the distribution of UMB4721 profiled by DASL appeared similar to UMB4849, and the scatterplot showed adherence to the y = x line. RIN of tissue sample or reference sample are listed in the histogram box. Cor = correlation.

High direct correlations were also achieved using the DASL-based assay between detected genes of intact reference brain RNA and degraded reference brain RNA samples only (Figures 2 and Additional File 2). Correlation with intact RNA yielded a correlation of 0.98 at 10 minutes, 0.92 at 30 minutes, and 0.82 at 60 minutes of degradation. Similarity between degraded and intact samples achieved by the IVT-based assay, however, was much lower. Correlations of 0.93 at 10 minutes, 0.75 at 30 minutes, and 0.5 at 60 minutes with intact RNA were found. In general, partial RNA degradation affected both assays (Additional File 3), but the IVT-based assay was affected more severely than the DASL-based assay (Figures 1 and 2, and Additional File 4) as evidenced by lower correlations of degraded reference samples with standard intact conditions.

### DASL-based platform is more reliable than IVT-based platform on RNA from postmortem frozen brain tissue but not formalin-fixed tissue

**Figure 3:**
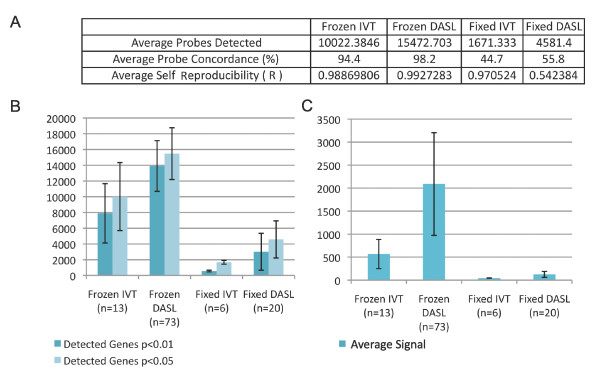
**Probe detection of two assays with RNA from frozen and fixed tissues**. (A) Summary of detected probes, probe concordance (detection p-value < 0.05 between technical replicates) and reliability of frozen and fixed tissue-based RNA profiling (correlation between technical replicates) on IVT- and DASL-based platforms are shown. DASL appears to confer some advantage over traditional IVT-based methods for tissue-based expression profiling. (B) Detected genes at p < 0.01 and p < 0.05 of frozen and fixed tissue-based RNA profiling on IVT- and DASL-based platforms. (C) Average signal detected from frozen and fixed tissue-based RNA profiling on IVT- and DASL-based platforms.

We next assessed the performance of DASL- and IVT-based assays on RNA extracted from postmortem frozen and formalin-fixed brain tissue (Table 1). Generally, average probes detected, average probe concordance, and average signal were higher for the DASL-based assay than the IVT-based assay for RNA extracted from frozen tissue and formalin fixed tissue (Figure 3). Average self-reproducibility was also higher for the DASL-based assay than for the IVT-based assay in frozen tissue, but not formalin fixed tissue. Upon examination of scatterplots of the formalin fixed genome-wide data, however, it was apparent that the high correlation between technical replicates in the IVT-based assay was due to detected expression only at background levels for these samples (Additional File 5).

**Table 1:** Frozen and formalin fixed tissue samples assayed

Case ID	Diagnosis	Age	Sex	COD	PMI	RIN	Frozen/Fixed
B6399	Autism	2	M	Drowning	4	6	Frozen

UMB4671	Autism	4	F	Accident, multiple injuries	13	7.7	Frozen

B1469	Autism	5	M	Unknown	42.8	2.3	Frozen

B5569	Autism	5	M	Asphyxia due To Drowning	25.5	2.5	Frozen

UMB1349	Autism	5	M	Drowning	39	-	Frozen

UMB1174	Autism	7	F	Seizure, Hypotension	14	-	Frozen

UMB4849	Autism	7	M	Drowning	20	6.3	Frozen

B5666	Autism	8	M	Sarcoma	22.2	5	Frozen

UMB4231	Autism	8	M	Drowning	12	-	Frozen

UMB4721	Autism	8	M	Drowning	16	2.4	Frozen

UMB1182	Autism	9	F	Smoke Inhalation	24	-	Frozen

B4925	Autism	9	M	Seizure Disorder	27	2.2	Frozen

UMB797	Autism	9	M	Drowning	13	-	Frozen

UMB4899	Autism	14	M	Drowning	9	-	Frozen

B7079	Autism	15	M	Asphyxia	23	5.7	Frozen

B5223 (M1106)	Autism	16	M	Stopped Breathing	47.9	-	Fixed

B6184	Autism	18	F	Seizures	7	3.6	Frozen

B5144	Autism	20	M	Auto Trauma	23.7	-	Frozen

B6337	Autism	22	M	Aspirated on vomit/Seizure	25	-	Frozen

B5000	Autism	27	M	Drowning	8.3	-	Frozen

B6994	Autism	28	M	Seizures	43.25	3	Frozen

B6640	Autism	29	F	Seizures	17.83	-	Frozen

B5173	Autism	30	M	GastroIntestinal Bleeding, seizures	20.3	-	Frozen

B6677	Autism	30	M	Congestive heart failure	16	-	Frozen

B6401	Autism	39	M	Cardiac Tamponade	14	2.3	Frozen

UMB1445	Autism	45	M	Complications of ALS/Autism	23	-	Frozen

B7085	Autism	49	F	Colorectal cancer spread through abdomen	21	3.4	Frozen

B7109	Autism	51	M	Myocardial infarction	22	4	Frozen

B4498	Autism	56	M	Anoxic Encephalopathy	20	-	Frozen

B6736	Control	4	F	Acute bronchipneumonia after tonsillectomy	17	6.3	Frozen

UMB1499	Control	4	F	Lymphocytic Myocarditis	21	6.5	Frozen

UMB1185	Control	4	M	Drowning	17	2.1	Frozen

UMB4670	Control	4	M	Commotio Cordis	17	2.2	Frozen

UMB1377	Control	6	F	Drowning	20	-	Frozen

UMB1500	Control	6	M	Multiple Injuries	19	1.8	Frozen

UMB4898	Control	7	M	Drowning	12	5.1	Frozen

UMB1674	Control	8	M	Drowning	36	-	Frozen

UMB1860	Control	8	M	Cardiac arrythmia	5	-	Frozen

UMB1407	Control	9	F	Seizure, Asthma	20	5.7	Frozen

UMB1650	Control	10	M	Sudden Unexpected Death	24	2.1	Frozen

UMB1714	Control	12	M	Cardiac arrythmia	22	2.5	Frozen

UMB4787	Control	12	M	Asthma	15	6.4	Frozen

UMB1670 (M806)	Control	13	M	Asphyxia By Hanging	5	-	Fixed

UMB4722	Control	14	M	MVA Multiple Injuries	16	1.8	Frozen

UMB4638	Control	15	F	Chest Injuries	5	-	Frozen

B6207	Control	16	M	Heart attack/disease	26.2	-	Frozen

B6756	Control	16	M	Myocardial infarction	22	-	Frozen

UMB1796 (EC6)	Control	16	M	Multiple Injuries	16	-	Frozen and Fixed

B5251	Control	19	M	Pneumonia/respiratory infection	19	3.5	Frozen

UMB1649	Control	20	M	Multiple Injuries	22	4.9	Frozen

BTB3960	Control	25	F	Gunshot to the Chest	26	-	Frozen

UMB818	Control	27	M	Multiple Injuries	10	1.9	Frozen

B5873	Control	28	M	Unknown	23.3	-	Frozen

B5334	Control	30	M	Asphyxia	14.83	5.7	Frozen

B5352	Control	31	M	Asphyxia	33	3.8	Frozen

B5813	Control	41	M	Unknown	27	5.2	Frozen

BTB-3859 (EC5)	Control	44	M	Unknown	30	-	Fixed

B6208	Control	50	F	Heart attack/disease	20	-	Frozen

B4756	Control	56	M	Myocardial infarction	23	5.9	Frozen

B6860	Control	56	M	Unknown	22	6.3	Frozen

Case ID, diagnosis, age, gender, cause of death (COD), postmortem interval (PMI), RNA Integrity Number (RIN), and preservation method of the postmortem human brain samples assayed in this study are listed.

Through examining a small number of samples assayed on both IVT-based and DASL-based platforms, we observed that the IVT-based assay was more severely affected by RNA degradation than the DASL-based assay with RNA extracted from brain tissues also, in accordance with the reference RNA experiments (Figure 2). For example, though correlations between detected probes decreased with decreasing RIN in both assays, correlation between a sample with RIN 6.3 and a sample with RIN 2.4 was 0.84 in DASL-based assay, but only 0.14 in IVT-based assay. With few exceptions, correlations between the same degraded samples and relatively intact RNA samples from frozen tissue and reference RNA were higher between those assayed by the DASL-based assay than by IVT-based assay. In addition, distributions of detected probes were clearly affected for IVT-based profiling of samples with low RIN, but this was not the case in DASL-based profiling (Additional File 6). In general, correlations of detected probes from the same samples between platforms were low (Additional File 7). Correlations of samples from the same case (UMB1796), frozen and formalin fixed, within and between platforms were also low (Additional File 8).

Nonetheless, neither platform appeared to produce reliable results using RNA from formalin-fixed brain tissue (Additional Files 5 and 8). Few genes across samples were detected above baseline on either platform, but a subset of probes detected above baseline levels in these samples on the DASL-based assay may show some reproducibility (Additional File 5).

### Predictors of variance in tissue dataset

**Figure 4:**
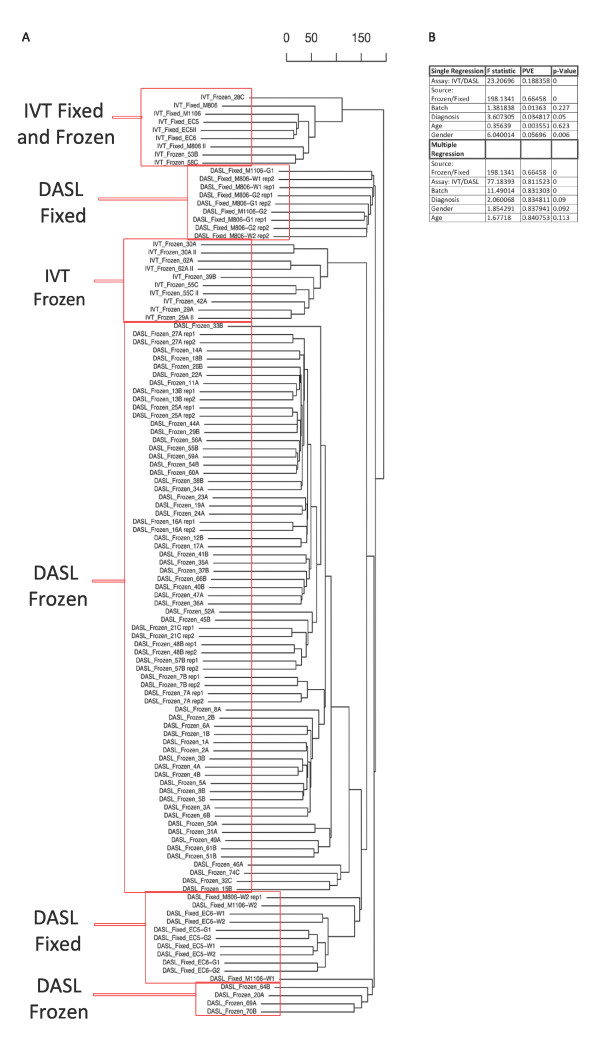
**Sources of dataset variance**. (A) Cluster dendrogram of average linkage showing similarities between assays and tissue types. Samples tended to cluster by assay type (IVT/DASL) and preservation style (Frozen/Fixed). (B) To quantify the effects of these predictors on the variance in the dataset, MDMR analysis was conducted. Single and multiple regression MDMR analysis are shown. Assay (IVT/DASL), source (Frozen/Fixed), batch, diagnosis, age, and gender of case from which tissue samples were taken were assessed. In the single regression model, each predictor is assessed separately; in the multiple regression model, each predictor is tested in relation with the other predictors in the model, yielding a cumulative percent variance explained (PVE).

We next analyzed variance prediction to understand important experimental and subject factors of gene expression differences in our dataset. These analyses can help determine statistical preprocessing that must be performed to prepare the dataset for differential expression analysis, and can indicate experimental considerations for future experiments. Hierarchical clustering by average linkage showed grouping of samples by assay type (DASL/IVT) and tissue preservation (frozen/formalin fixed; Figure 4A).

To quantify these and other predictors of variance in the dataset, we performed Multivariate Distance Matrix Regression (MDMR) analysis [14]. Multiple regression MDMR (Figure 4B) showed that tissue preservation method (frozen or fixed) accounted for 27% of the variance in the dataset, followed, in significance, by assay type [IVT or DASL; cumulative percentage of variance explained (PVE) = 10%], batch (PVE = 26%), age of the case (PVE = 0.9%), diagnosis (PVE = 0.8%), gender (PVE = 0.5%), and postmortem interval (PVE = 0.3%).

In a small sample of cases with available RIN (N = 4) and assayed on both IVT- and DASL-based assays, RIN also explained a large percentage of variability in the expression data. However, the influence of RIN on the IVT-processed samples (PVE = 82.45%, p = 0.21) in single regression MDMR was greater than that on the DASL-processed samples (PVE = 64.52, p = 0.1178). These results confirm that the IVT-based assay is more greatly influenced by differences in RNA degradation than the DASL-based assay.

### Validation of microarray results by qPCR

QPCR validation of the DASL-based microarray data was performed on a subset of genes. We compared the log2 fold changes of the qPCR data with the log2 transformed, quantile normalized data. Using a Spearman's rank correlation, the log2 fold changes of these 19 genes (Table 2) across qPCR and DASL-based microarray platforms were found to be correlated at R = 0.78 (p = 0.000075, DF = 17; Additional File 9).

**Table 2:** Primer sequences for RTPCR validation.

Gene Name	Forward Primer Sequence	Reverse Primer Sequence
AIF1	CTCCAGCTTGGTCTGTCTCC	TCATCCAGCCTCTCTTCCTG

CACNB1	ACGTCCTCGGATACCACATC	CGGTCCTCCTCCAGAGATAC

CASP10	CTTTGGACCTTGGAGCACAC	GAACTGGAATACCAATGTTGACC

CTTN	GAAACAGGACCAAAAGCTTCC	CATCTGGACACCAAACTTGC

EDEM3	GAATTTGAAGATGCAGTGAGAAAA	AACTGCTTTGCCATTTGGAG

HAP1	GATGGAGGAGAACAGCAAGC	GAATCTGAGTAGAGCTGGAGGAG

IL12RB1	CTGCCTGCAGAACCAGTGAG	CAGCTGTGGGACCCTCATAC

LAMA2	TGTTTCTGTTCAGGGGTTTCA	TGCTGATCTGCTGAGGTGAG

MTNR1B	TCTTGGTGAGTCTGGCATTG	TTGAAGACAGAGCCGATGAC

NEUROG2	CAAAGTCACAGCAACGCTGA	GAGCAGCACTAACACGTCCTC

NFKB2	CCCTCCCATGGAGGACTG	ACCAGACTGTGGGCATGAG

NRXN1	TCAGGAAATTCGCTTTGACC	GTGTTGGTGATGCATTTTGG

OPN4	ACCCAGCTGGTGGGACAG	CTGTGCCCAGGGTATAGTGG

OR2B6	TGAATTGGGTAAATGACAGCA	CATGGGGGTATGAAGTTTGG

PROK1	CACCCCAAGTGACCATGAG	CTCGAAGCCACAGGCTGAT

SRC	AGATCCGCAAGCTGGACA	CTGAGTCTGCGGCTTGGAC

TBX1	GTGTGAGCGTGCAGCTAGAG	TCCATGAGCAGCATATAGTCG

TGFA	CCTTGGTGGTGGTCTCCAT	CGGTTCTTCCCTTCAGGAG

TLR1	AGGCCCTCTTCCTCGTTAGA	AATGGCAAAATGGAAGATGC

RPL13A	GGGAAGGGTTGGTGTTCAT	GGGAAGGGTTGGTGTTCAT

ACTB	GCCGTCTTCCCCTCCATC	CGTCCCAGTTGGTGACGAT

TBP	CGGCTGTTTAACTTCGCTTC	CCAGCACACTCTTCTCAGCA

Forward and reverse primer sequences for 19 experimental and 3 reference genes used for RT-PCR to validate microarray results are shown.

## Discussion

Recent advances in gene expression technology have made genome-wide expression profiling possible in partially degraded RNA samples [5,7]. DASL technology has been applied reliably to even formalin fixed, paraffin-embedded tissues from a range of tissue sources [8,10-13]. It has, however, not yet been utilized to profile expression in brain tissue, in which RNA degradation levels are known to be high and availability is limited [1-3]. We have shown that the DASL-based genome-wide expression-profiling approaches applied to partially degraded brain-specific reference RNA and postmortem brain tissue-extracted RNA may confer some advantages over traditional IVT-based methods. Our observations raise questions about the reliability of the assays that could impact interpretation of association analyses involving gene expression levels.

The greater the degradation of RNA, the less reliable results from IVT- and DASL-based platforms become. Therefore, although the DASL-based approach appeared to recover more reliable gene expression values from partially degraded RNA samples, reliable results may still not be gleaned from the most severely degraded and chemically modified RNA samples such as from formalin-fixed brain tissue. The main cause of failure using DASL in formalin-fixed brain tissue may be difficulty with performing the reverse transcription step. Unfortunately, even though the DASL-based assay performed better than the IVT-based assay in limited brain frozen tissues, our sample sizes were too small to make conclusive arguments about the relative efficacy of DASL- and IVT-based platforms on RNA extracted from frozen and formalin fixed tissues. Our results, however, suggest that the DASL-based platform may confer some advantages for profiling partially degraded RNA from frozen brain tissue.

Through the examination of the expression datasets in tissue-based RNA samples from control and autistic cases, we explored important factors to consider in postmortem brain tissue expression profiling. The most important explanatory variable of gene expression profile variance across samples in our dataset was in fact not the platform on which the RNA was assayed, but how the tissue was preserved. This result has important implications for brain banks in the methods used for preserving RNA [15] and for interpreting and comparing across brain gene expression studies, especially in neuropsychiatric disorders [1] with a spectrum of phenotypes like autism. Though in general the DASL-based approach is more reliable than the IVT-based approach for profiling degraded mRNA samples, our MDMR analyses suggest that RNA degradation and other factors still play important roles in determining dataset variance, thereby potentially confounding differential expression analyses. These additional factors may be dealt with during data preprocessing steps using such statistical tools as ComBat [16], and is described in Chow and Pramparo et al.: Early brain gene expression and copy number anomalies in autism, submitted.

## Conclusions

Nonetheless, the study of neuropsychiatric disorders may benefit from DASL-based expression profiling technology, especially when investigating molecular pathways involved in diseases that cannot be modeled by animals [4]. This platform and other expression profiling methods will be vital in helping make use of scarce and precious brain tissue to elucidate uniquely human genetic pathogenic mechanisms.

## Methods

### Artificial Degradation of Reference RNA

Ambion^® ^human reference brain RNA and Stratagene^® ^reference pooled RNA samples were heated at 95°C for 0, 10, 30, and 60 minutes to artificially degrade them. Samples were analyzed using BioAnalyzer^® ^(Agilent Technologies) to visualize levels of degradation (Additional File 1). Combinations of 75% brain reference RNA and 25% pooled reference RNA, and 75% pooled reference RNA and 25% brain reference RNA were also mixed for comparison with pure samples. Samples were then prepared for profiling on microarray platforms as described below.

### Frozen and formalin-fixed postmortem human brain samples for gene expression profiling

57 frozen blocks of fresh frozen brain tissue and 4 blocks of formalin-fixed brain tissue from the prefrontal cortex of controls and autistic, male and female cases were obtained from the Harvard Brain and Tissue Resource Center (United States Public Health Service) and from the University of Miami/University of Maryland Brain and Tissue Bank (National Institute of Child Health and Human Development; Table 1).

Diagnostic criteria of Autistic Disorder was verified for all autistic cases by review of psychological and medical records, including the Autism Diagnostic Interview-Revised ([17]; ADI-R), and the Autism Diagnostic Observation Schedule ([18]; ADOS) by a psychologist with extensive diagnostic experience with autism (CCB; Table 1). Seizure incidence of autistic cases was also assessed through case records.

All cases were deceased, and were deidentified by the brain banks where tissue was obtained. However, the same human protections procedures were employed as for live subjects. Research procedures employed in this study were approved by the institutional review board of the University of California, San Diego (protocol number 091205).

### Brain sample collection

Due to documented variability of gene expression in neighboring brain areas [19,20], it is of extreme importance that the blocks of tissue chosen for gene expression profiling are from comparable regions between cases. Anatomical landmarks were identified as consistently as possible for dissection across cases with the goal of obtaining a set of highly controlled, comparable tissue for brain gene expression profiling. When available, tissue from the superior frontal gyrus of the dorsal lateral prefrontal cortex (DLPFC) was dissected in each case. When this area was not available, we sampled from the middle frontal gyrus. The formalin-fixed samples were obtained from larger areas of frontal cortex. Cytoarchitecture and anatomical landmarks were also used to determine the area of DLPFC similar to that of the frozen tissue for dissection.

### RNA Extraction from Tissues

Extraction of total RNA from 5-10 mg of frozen tissue from both grey and white matter, with as many layers of cortex as possible, was performed using MELT^® ^kit from Ambion according to manufacturer's instructions (http://www.ambion.com). Extraction of total RNA from 5-10 mg of formalin-fixed tissue sections was performed using the Roche^® ^High Pure FFPE RNA Micro Kit. Select RNA samples were analyzed with BioAnalyzer^® ^(Agilent) according to the manufacturer's protocol for quality control and quantification, and available RNA Integrity Numbers (RIN) are reported in Table 1. Whole RNA from remaining samples was quantified using a NanoDrop^® ^spectrophotometer.

### DASL Labeling, Hybridization, and Scanning

Total RNA from reference samples, frozen, and formalin-fixed cases underwent cDNA synthesis, and cDNA-mediated annealing, selection, and ligation (DASL)-based labeling, hybridization to Illumina HumanRef8 v3 and Human 12K microarrays (DASL assay on reference RNA samples only), and scanning on two separate occasions as described previously [7]. Both biological and technical replicates were included for quality control. Using biotinylated random primers and oligo-dT, 200 ng RNA was converted to cDNA. The biotinylated cDNA was then immobilized to a streptavidin-coated solid support, and annealed with a pool of gene-specific oligonucleotides.

Following extension and ligation, the ligated oligonucleotides were PCR amplified with a biotinylated and a fluorophore-labeled universal primer, and captured using streptavidin paramagnetic beads. Finally, the single-stranded PCR products were eluted and hybridized to the BeadChips at 58°C for 16 hours. A BeadArray Reader was used to scan array images and extract fluorescence intensities, and all data were uploaded into BeadStudio software without normalization or background subtraction for quality control and processing. All raw data is available on the NCBI Gene Expression Omnibus under accession number GSE28475 (http://www.ncbi.nlm.nih.gov/geo/).

### IVT Labeling, Hybridization, and Scanning

Gene expression profiling was performed on RNA from reference samples, frozen, and fixed cases using the Illumina Human Ref-8 v3 Expression BeadChip platform (Illumina Inc., San Diego, CA, USA) according to manufacturer's protocols. Following RNA extraction, an IVT reaction for biotinylated cRNA was performed overnight (~16 h). 750 ng cRNA were hybridized on the beadchip at 58°C overnight and detected with Cyanine3-streptavidin. Arrays were again scanned with the Illumina BeadArray Reader and read into Illumina GenomeStudio software without normalization or background subtraction.

### Microarray data analysis

All data analyzed were raw and unprocessed. Probe detection and signal information was directly output from GenomeStudio. Probe concordance and self-reproducibility were calculated based on technical replicates in each category (frozen tissue-RNA assayed by IVT, frozen tissue-RNA assayed by DASL, fixed tissue-RNA assayed by IVT, fixed tissue-RNA assayed by DASL).

All plots were generated using the R/Bioconductor package Lumi [21-23] and Microsoft Excel. Cluster dendrograms were generated by Lumi using Euclidean distance and average linkage clustering.

#### Multivariate Distance Matrix Regression

To assess the variance within the dataset attributable to a set of variables before and after manipulating and pre-processing the expression assay results (e.g. batch correction), multivariate distance matrix regression (MDMR; [14]) with 1000 permutations was applied to the Euclidean distance matrices constructed from the expression values between each sample (http://polymorphism.scripps.edu/~cabney/cgi-bin/mmr.cgi). Variables of interest that were related to the expression profiles reflected in the distance matrices included batch, RNA source (reference RNA, frozen tissue, formalin-fixed tissue), assay type (DASL or IVT), gender, diagnosis, and age of cases from which we sampled. We leveraged both single independent variable and multiple independent MDMR results. Case data analyzed by MDMR as predictors (diagnosis, age, seizures) were compiled by a clinical psychologist (C.C.B).

### Independent qPCR Validation of Microarray Results

#### Genes and Cases

RNA from 1 male autistic and 1 male control case of 31 years was analyzed using SYBR green RT-PCR to validate the intensity values detected by microarray. 19 genes were chosen with a wide range of fold change values (positive and negative), and are listed in Table 2. Using Primer3 software [24], primers of these genes were designed across splice junctions to avoid artifacts by genomic DNA contamination and to produce amplicons of ~200 bp. RPL13A, B2M and ACTB, three genes highly expressed in the brain at stable levels [25] were chosen as reference genes for each experiment. Expression values for the remainder of the genes were normalized to these reference gene controls.

#### cDNA Synthesis and qPCR

One microgram of total RNA was used for cDNA synthesis using random hexamers and AMV reverse transcriptase. An equivalent of 50 ng of RNA was processed by qPCR using Roche's LightCycler rapid thermal cycler system (Roche Diagnostics Ltd, Lewes, UK) according to the manufacturer's instructions, in a 96-well, 10-uL format using standard PCR conditions. 1 μL of cDNA template, 250 nM of forward and reverse primer, and 5 μL of qPCR Master Mix (Roche) were mixed for each reaction.

### Statistical analysis

According to Vandesompele et al., [25] we took the geometric mean of all reference genes and the difference between this mean and the average intensity of experimental genes to find the delta Ct for each experimental gene. Subsequently, log2 fold change was assessed using -(T-C) where T = delta Ct of gene of the autistic case, and C = delta Ct of gene of the control case. Spearman's rank correlation was then applied to the results from the qPCR and microarray assays.

## Authors' contributions

MLC participated in designing and conducting experiments, data analysis, interpretation of results, and constructing the manuscript. HRL participated in designing and conducting experiments, and revising the manuscript. MEW participated in data analysis. CCB performed clinical assessments on autopsy reports to collect case data for data analysis and revised the manuscript. CA and JBF participated in microarray processing. JBF also revised the manuscript. AWB, XDF and EC designed and supervised experiments. NJS designed and supervised experiments, interpreted results, and revised the manuscript. Each author has given final approval to the manuscript for publication.

## Supplementary Material

Additional File 1 **BioAnalyzer analysis showing reference RNA fragmentation and RIN values for each sample**. Artificially degraded brain and pooled reference RNA was visualized using Agilent BioAnalyzer. B0 and P0 samples show two ribosomal RNA bands, while samples heated at 95°C showed smaller RNA fragments. The longer the duration of heating, the smaller the fragments became. B0 = intact reference brain RNA; B10 = 10 minute heating at 95°C; B30 = 30 minute heating at 95°C; B60 = 60 minute heating at 95°C; P0 = intact reference pooled RNA; P10 = 10 minute heating at 95°C; P30 = 30 minute heating at 95°C; P60 = 60 minute heating at 95°C. Click here for file

Additional File 2 **Brain Reference RNA assay performance**. Scatterplots of reference RNA samples with increasing levels of artificial degradation, histogram of data distribution, correlation, and number of up- and downregulated genes differing between samples in IVT- (left) and DASL- (right) based platforms are shown. Degraded brain reference RNA profiled on IVT-based assays show lower correlations with intact RNA than on DASL-based assays. Intensity values also cluster at background levels for increasing RNA degradation for IVT-based samples but not DASL-based samples. For example, correlations between intact RNA and RNA degraded for 60 minutes (B60) differed greatly between two assays (four corner boxes on the two plots). IVT_B60 had a correlation of 0.5 (lower left corner box) with the intact sample (IVT_B0). In contrast, DASL_B60 had a correlation of 0.82 with the intact sample (DASL_B0). In addition, the histogram of IVT_B60 shows most intensity values at background levels, but that of DASL_B60 showed a similar distribution to that of DASL_B0. In addition, the scatterplot comparing IVT_B0 and IVT_B60 (top right corner box) shows skewing from the y = x line, reflecting the clustering of IVT_B60 intensities at background values. The scatterplot comparing DASL_B0 and DASL_B60, however, shows better adherence to the diagonal despite some dispersion. B0 = intact reference brain RNA; B10 = 10 minute heating at 95°C; B30 = 30 minute heating at 95°C; B60 = 60 minute heating at 95°C. Click here for file

Additional File 3 **Clustering of samples with similar levels of RNA degradation evident in results from both IVT and DASL assays**. Average hierarchical clustering was applied to the Euclidean distance between intact and artificially degraded brain and pooled reference RNA samples. Thus the more degraded the sample is regardless of assay, the less similar it is to the intact sample. Labels indicate assay type and time of degradation. B0 = intact reference brain RNA; B10 = 10 minute heating at 95°C; B30 = 30 minute heating at 95°C; B60 = 60 minute heating at 95°C; P0 = intact reference pooled RNA; P10 = 10 minute heating at 95°C; P30 = 30 minute heating at 95°C; P60 = 60 minute heating at 95°C; B75P25 = 75% brain reference RNA and 25% pooled reference RNA combination; B25P75 = 25% brain reference RNA and 75% pooled reference RNA combination; rep = technical replicates. Click here for file

Additional File 4 **Intensity distributions vary with RNA degradation in IVT- but not DASL-based assays**. Boxplot showing the amplitude of genome-wide intensity distributions detected for intact and artificially degraded reference brain and pooled RNA samples processed by IVT- and DASL-based platforms. In the IVT-processed samples, increasing RNA degradation correlated with a smaller range of intensity values clustered at low intensities. In the DASL-processed samples, this confound was not observed. B0 = intact reference brain RNA; B10 = 10 minute heating at 95°C; B30 = 30 minute heating at 95°C; B60 = 60 minute heating at 95°C; P0 = intact reference pooled RNA; P10 = 10 minute heating at 95°C; P30 = 30 minute heating at 95°C; P60 = 60 minute heating at 95°C; B75P25 = 75% brain reference RNA and 25% pooled reference RNA combination; B25P75 = 25% brain reference RNA and 75% pooled reference RNA combination; rep = technical replicate. Click here for file

Additional File 5 **Assay performance on formalin-fixed tissue**. Correlations between fixed tissue technical replicates and between samples on IVT- (left) and DASL- (right) based platforms are shown. Scatterplots of genome-wide profiling results from 2 formalin-fixed samples (BTB3859 and UMB1670) processed by the two assays, histogram of data distribution, correlation, and number of up and downregulated genes show low reliability in profiling RNA from these samples in both assays. Most intensity values tended to cluster at background levels, particularly for the IVT-processed samples. For example, technical replicates of BTB3859 (four top left boxes on two plots) show 0.83 correlation on the IVT-based assay, and 0.77 correlation on the DASL assay. However, the histogram and scatterplots show that this correlation on the IVT-based assay (left plot) is due to detection of most probes at low background levels. In contrast, the histogram of the DASL-based assay results for this case (right plot) shows a subset of probes that showed some reliability, with intensity values above baseline. Rep = technical replicate. Click here for file

Additional File 6 **RNA degradation in degraded frozen-tissue based RNA affects IVT greater than DASL processing**. Boxplots show genome-wide intensity distribution of frozen brain-extracted RNA samples from four cases and replicates processed by IVT- and DASL-based assays. The IVT-processed samples clearly show distributions clustered at background intensities especially for samples with low RIN (2.2 for UMB4670 and 2.4 for UMB4721). The distribution of DASL-processed samples for UMB4670 and UMB4721 did not appear significantly different from samples with higher RIN (6.3 for UMB4849 and 4.9 for UMB1649). Rep = replicate. Click here for file

Additional File 7 **Low correlations between IVT- and DASL-based assays on the same three frozen tissue-extracted RNA samples**. Scatterplots of 3 samples processed by the two assays, histogram of data distribution, correlation, and number of up and downregulated genes show low concordance between the same tissue sample processed by two platforms, and differences in intensity distribution between platforms. For example, the correlation between case UMB1349 processed by IVT- and DASL-based assays (IVT_UMB1349 and DASL_UMB1349; four top left boxes) was 0.76. Histograms for this case show a similar distribution for both assays, but the scatterplot shows many probes diverging from the y = x line. Click here for file

Additional File 8 **Low correlation between processed samples of UMB1796**. Frozen and fixed samples taken from case UMB1796 were processed by both IVT- and DASL-based methods. Genome-wide results are plotted to compare correlations and distributions. Scatterplots of UMB1796 replicates, histogram of data distribution, correlation, and number of up- and downregulated genes differing between samples show low correlations between brain tissue profiled using IVT- and DASL-based approaches from the same case in all except DASL technical replicates. Labels indicate assay type (DASL/IVT), preservation method (Frozen/Fixed), sample code, and replicate number (if applicable). Click here for file

Additional File 9 **Log2 Fold Change Correlations of selected genes detected by microarray and RTPCR**. Log2 Fold change detected by RTPCR is depicted on the x axis, and microarray on the y-axis of 19 up- and down-regulated genes in the dataset. Spearman's rank correlation detected an R = 0.78 (p = 0.000075, DF = 17) correlation between microarray and qPCR detection of fold change. Click here for file
