# Supplementary figures and images for: Genome-wide expression assay comparison across frozen and fixed postmortem brain tissue samples
Source: BMC Genomics. 2011 Sep 10;12:449. doi: 10.1186/1471-2164-12-449 (PMC3179967; doi:10.1186/1471-2164-12-449)

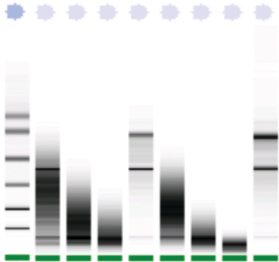

|        |     |     |     |     |     |     |     |     |
|--------|-----|-----|-----|-----|-----|-----|-----|-----|
| Sample | B10 | B30 | B60 | B0  | P10 | P30 | P60 | P0  |
| RIN    | 2.7 | 1.9 | 2.3 | 7.3 | 1.7 | 2.4 | 2.6 | 8.1 |

Supplement: Additional File 1 — BioAnalyzer analysis showing reference RNA fragmentation and RIN values for each sample. Artificially degraded brain and pooled reference RNA was visualized using Agilent BioAnalyzer. B0 and P0 samples show two ribosomal RNA bands, while samples heated at 95°C showed smaller RNA fragments. The longer the duration of heating, the smaller the fragments became. B0 = intact reference brain RNA; B10 = 10 minute heating at 95°C; B30 = 30 minute heating at 95°C; B60 = 60 minute heating at 95°C; P0 = intact reference pooled RNA; P10 = 10 minute heating at 95°C; P30 = 30 minute heating at 95°C; P60 = 60 minute heating at 95°C. [file 1471-2164-12-449-S1.PDF]

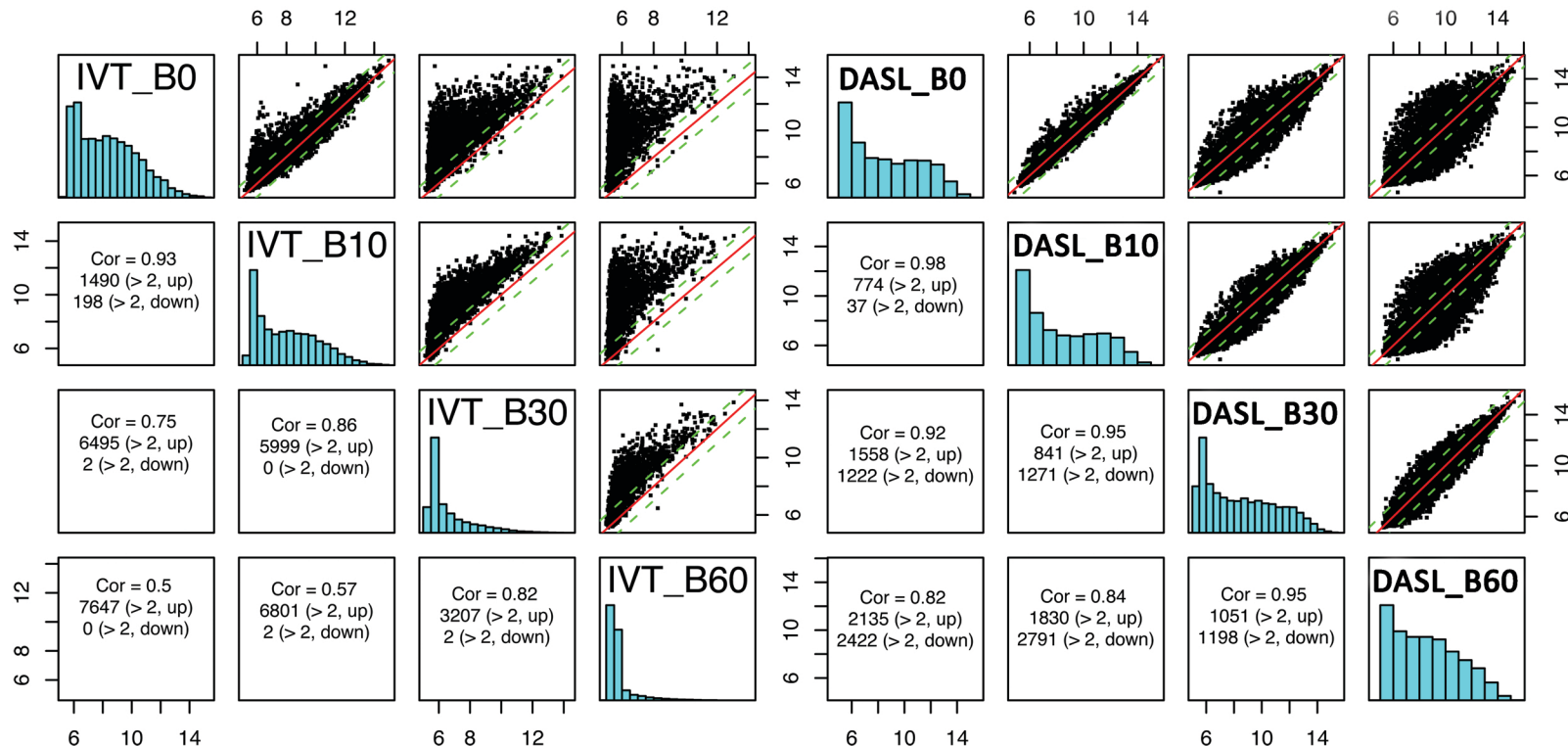

Supplement: Additional File 2 — Brain Reference RNA assay performance. Scatterplots of reference RNA samples with increasing levels of artificial degradation, histogram of data distribution, correlation, and number of up- and downregulated genes differing between samples in IVT- (left) and DASL- (right) based platforms are shown. Degraded brain reference RNA profiled on IVT-based assays show lower correlations with intact RNA than on DASL-based assays. Intensity values also cluster at background levels for increasing RNA degradation for IVT-based samples but not DASL-based samples. For example, correlations between intact RNA and RNA degraded for 60 minutes (B60) differed greatly between two assays (four corner boxes on the two plots). IVT_B60 had a correlation of 0.5 (lower left corner box) with the intact sample (IVT_B0). In contrast, DASL_B60 had a correlation of 0.82 with the intact sample (DASL_B0). In addition, the histogram of IVT_B60 shows most intensity values at background levels, but that of DASL_B60 showed a similar distribution to that of DASL_B0. In addition, the scatterplot comparing IVT_B0 and IVT_B60 (top right corner box) shows skewing from the y = x line, reflecting the clustering of IVT_B60 intensities at background values. The scatterplot comparing DASL_B0 and DASL_B60, however, shows better adherence to the diagonal despite some dispersion. B0 = intact reference brain RNA; B10 = 10 minute heating at 95°C; B30 = 30 minute heating at 95°C; B60 = 60 minute heating at 95°C. [file 1471-2164-12-449-S2.PDF]

Sample relations based on 2787 genes with sd/mean > 0.1

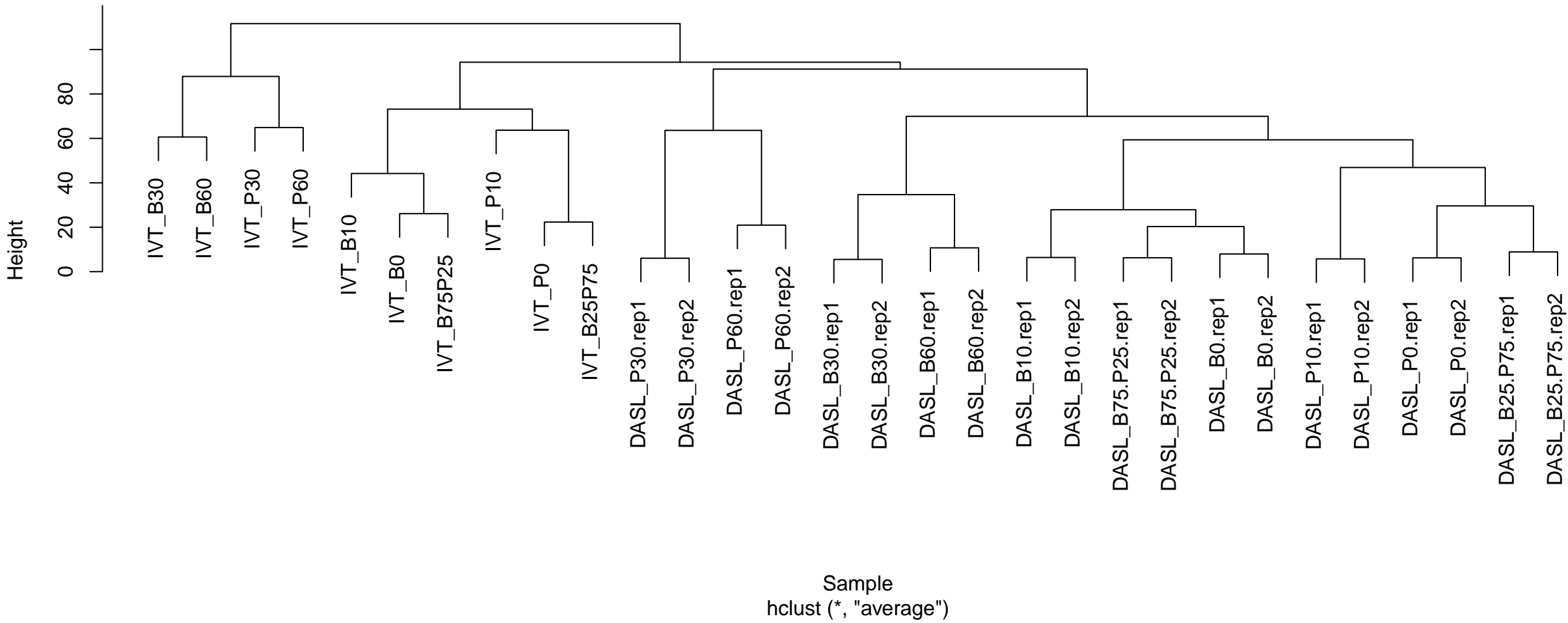

Supplement: Additional File 3 — Clustering of samples with similar levels of RNA degradation evident in results from both IVT and DASL assays. Average hierarchical clustering was applied to the Euclidean distance between intact and artificially degraded brain and pooled reference RNA samples. Thus the more degraded the sample is regardless of assay, the less similar it is to the intact sample. Labels indicate assay type and time of degradation. B0 = intact reference brain RNA; B10 = 10 minute heating at 95°C; B30 = 30 minute heating at 95°C; B60 = 60 minute heating at 95°C; P0 = intact reference pooled RNA; P10 = 10 minute heating at 95°C; P30 = 30 minute heating at 95°C; P60 = 60 minute heating at 95°C; B75P25 = 75% brain reference RNA and 25% pooled reference RNA combination; B25P75 = 25% brain reference RNA and 75% pooled reference RNA combination; rep = technical replicates. [file 1471-2164-12-449-S3.PDF]

Boxplot of microarray intensity

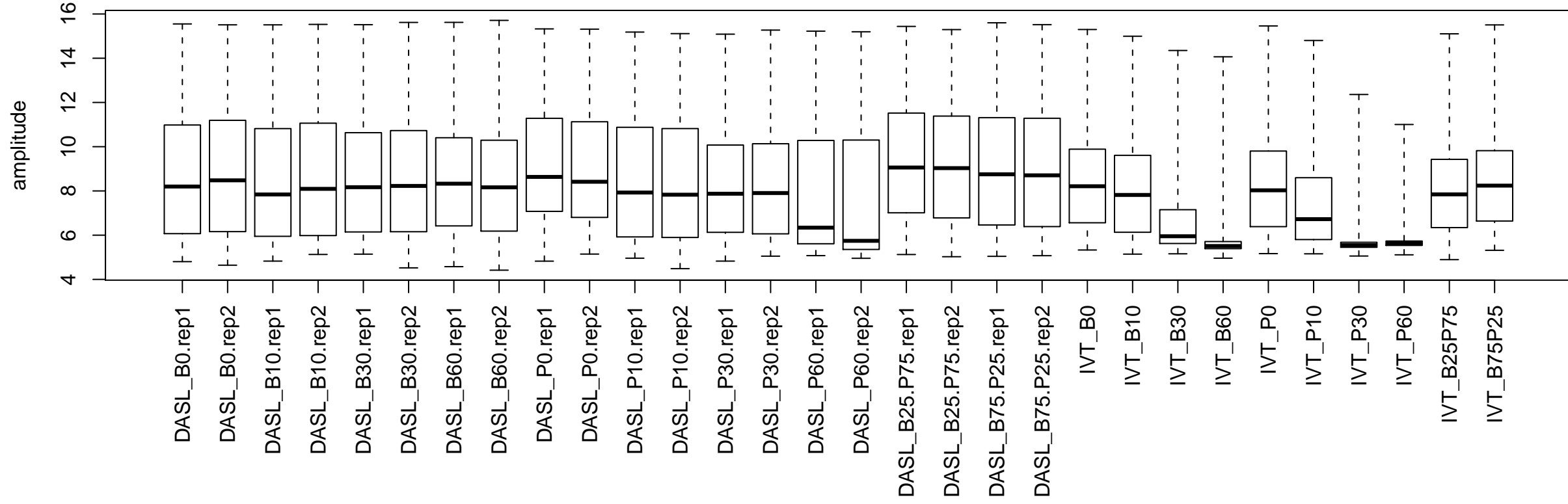

Supplement: Additional File 4 — Intensity distributions vary with RNA degradation in IVT- but not DASL-based assays. Boxplot showing the amplitude of genome-wide intensity distributions detected for intact and artificially degraded reference brain and pooled RNA samples processed by IVT- and DASL-based platforms. In the IVT-processed samples, increasing RNA degradation correlated with a smaller range of intensity values clustered at low intensities. In the DASL-processed samples, this confound was not observed. B0 = intact reference brain RNA; B10 = 10 minute heating at 95°C; B30 = 30 minute heating at 95°C; B60 = 60 minute heating at 95°C; P0 = intact reference pooled RNA; P10 = 10 minute heating at 95°C; P30 = 30 minute heating at 95°C; P60 = 60 minute heating at 95°C; B75P25 = 75% brain reference RNA and 25% pooled reference RNA combination; B25P75 = 25% brain reference RNA and 75% pooled reference RNA combination; rep = technical replicate. [file 1471-2164-12-449-S4.PDF]

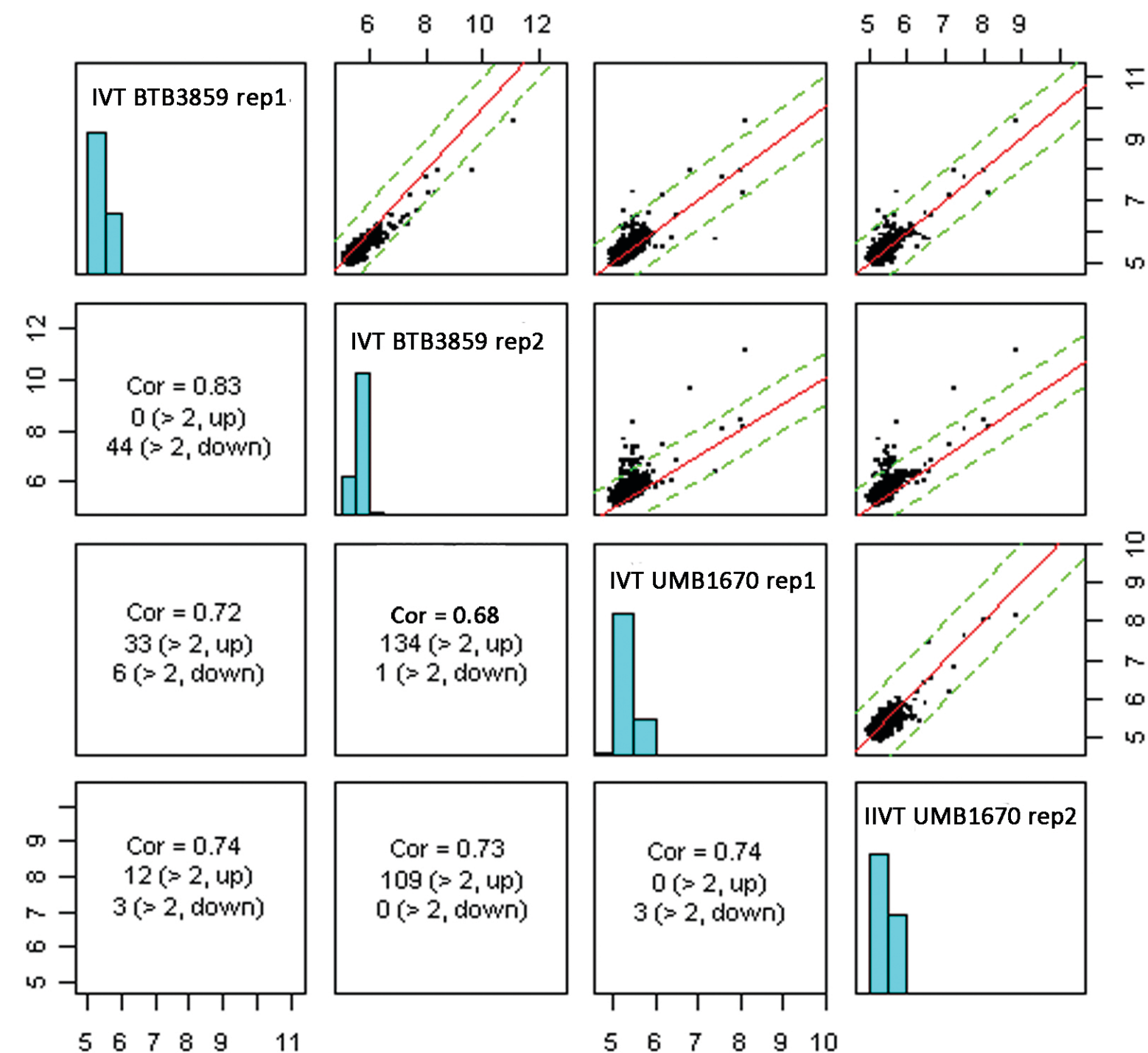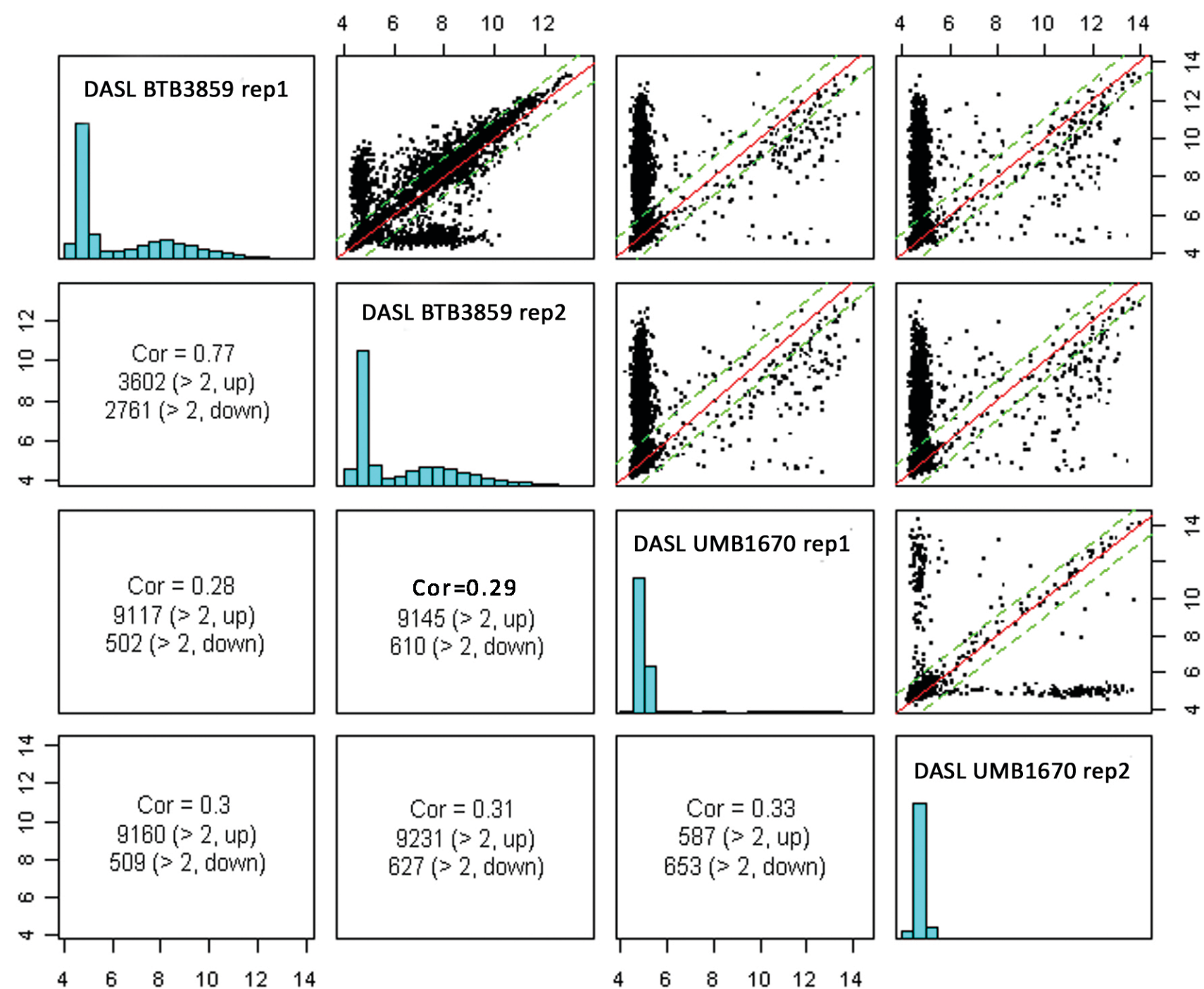

Supplement: Additional File 5 — Assay performance on formalin-fixed tissue. Correlations between fixed tissue technical replicates and between samples on IVT- (left) and DASL- (right) based platforms are shown. Scatterplots of genome-wide profiling results from 2 formalin-fixed samples (BTB3859 and UMB1670) processed by the two assays, histogram of data distribution, correlation, and number of up and downregulated genes show low reliability in profiling RNA from these samples in both assays. Most intensity values tended to cluster at background levels, particularly for the IVT-processed samples. For example, technical replicates of BTB3859 (four top left boxes on two plots) show 0.83 correlation on the IVT-based assay, and 0.77 correlation on the DASL assay. However, the histogram and scatterplots show that this correlation on the IVT-based assay (left plot) is due to detection of most probes at low background levels. In contrast, the histogram of the DASL-based assay results for this case (right plot) shows a subset of probes that showed some reliability, with intensity values above baseline. Rep = technical replicate. [file 1471-2164-12-449-S5.PDF]

**Boxplot of microarray intensity**

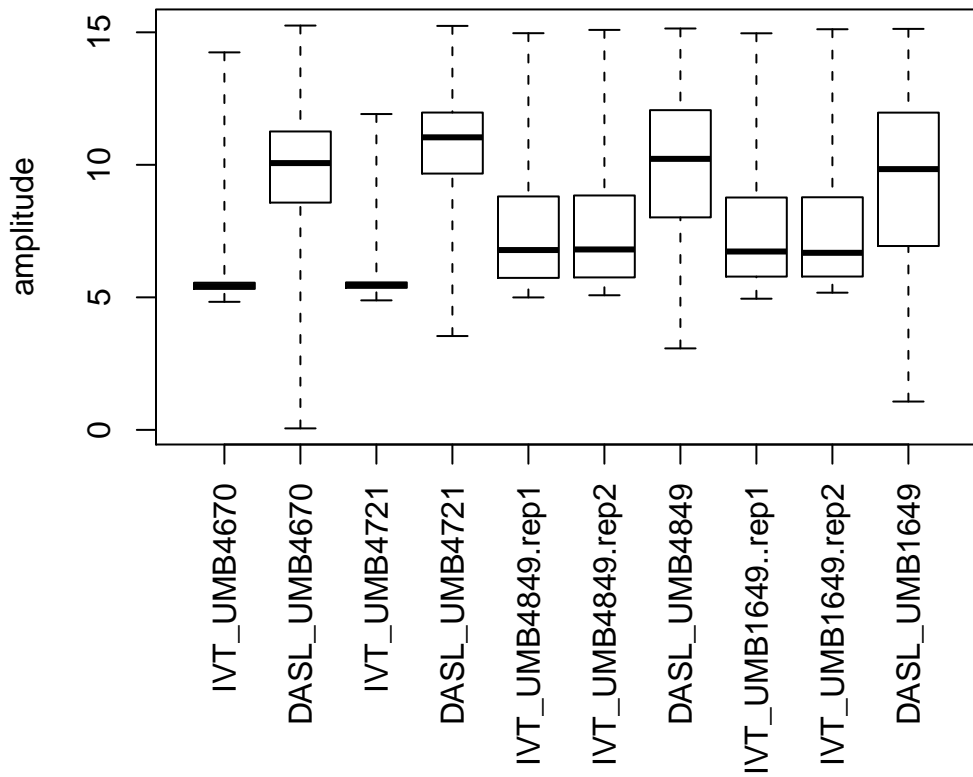

Supplement: Additional File 6 — RNA degradation in degraded frozen-tissue based RNA affects IVT greater than DASL processing. Boxplots show genome-wide intensity distribution of frozen brain-extracted RNA samples from four cases and replicates processed by IVT- and DASL-based assays. The IVT-processed samples clearly show distributions clustered at background intensities especially for samples with low RIN (2.2 for UMB4670 and 2.4 for UMB4721). The distribution of DASL-processed samples for UMB4670 and UMB4721 did not appear significantly different from samples with higher RIN (6.3 for UMB4849 and 4.9 for UMB1649). Rep = replicate. [file 1471-2164-12-449-S6.PDF]

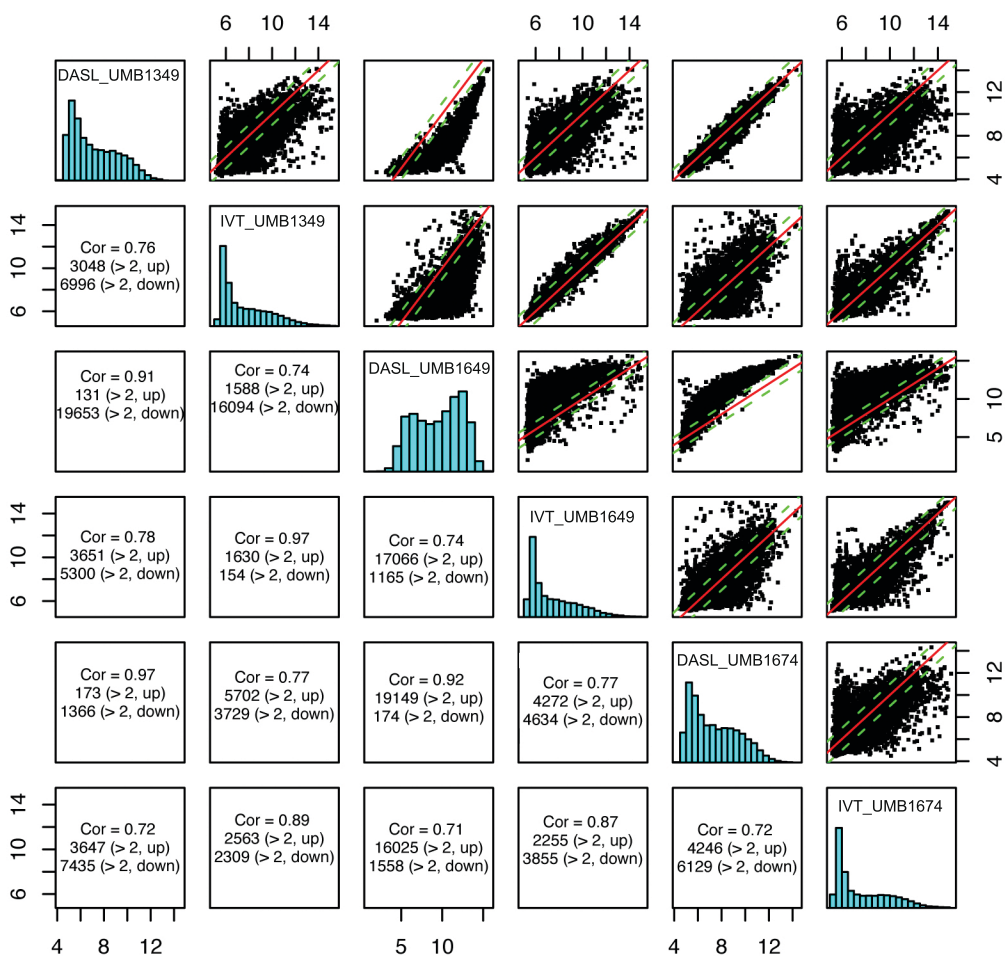

Supplement: Additional File 7 — Low correlations between IVT- and DASL-based assays on the same three frozen tissue-extracted RNA samples. Scatterplots of 3 samples processed by the two assays, histogram of data distribution, correlation, and number of up and downregulated genes show low concordance between the same tissue sample processed by two platforms, and differences in intensity distribution between platforms. For example, the correlation between case UMB1349 processed by IVT- and DASL-based assays (IVT_UMB1349 and DASL_UMB1349; four top left boxes) was 0.76. Histograms for this case show a similar distribution for both assays, but the scatterplot shows many probes diverging from the y = x line. [file 1471-2164-12-449-S7.PDF]

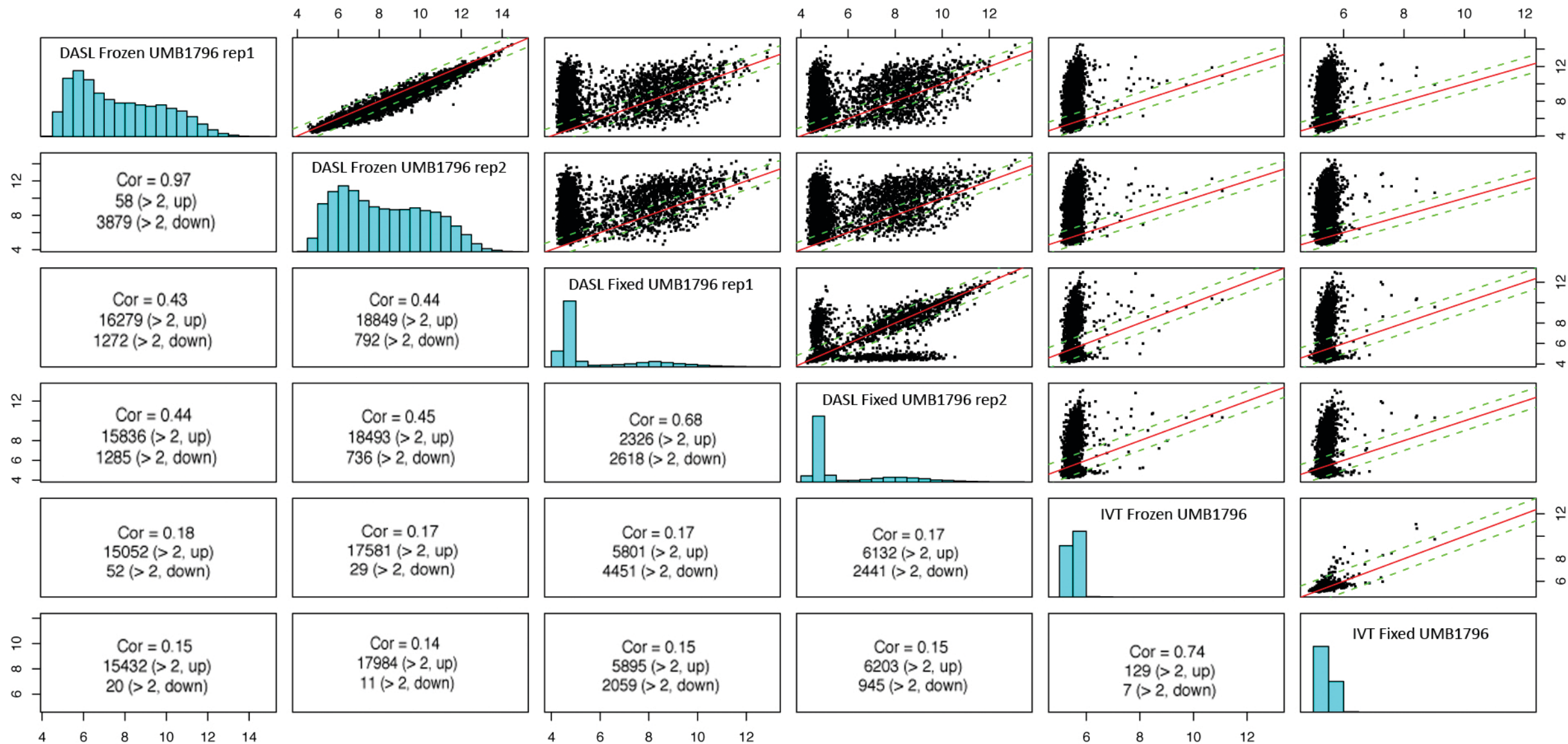

Supplement: Additional File 8 — Low correlation between processed samples of UMB1796. Frozen and fixed samples taken from case UMB1796 were processed by both IVT- and DASL-based methods. Genome-wide results are plotted to compare correlations and distributions. Scatterplots of UMB1796 replicates, histogram of data distribution, correlation, and number of up- and downregulated genes differing between samples show low correlations between brain tissue profiled using IVT- and DASL-based approaches from the same case in all except DASL technical replicates. Labels indicate assay type (DASL/IVT), preservation method (Frozen/Fixed), sample code, and replicate number (if applicable). [file 1471-2164-12-449-S8.PDF]

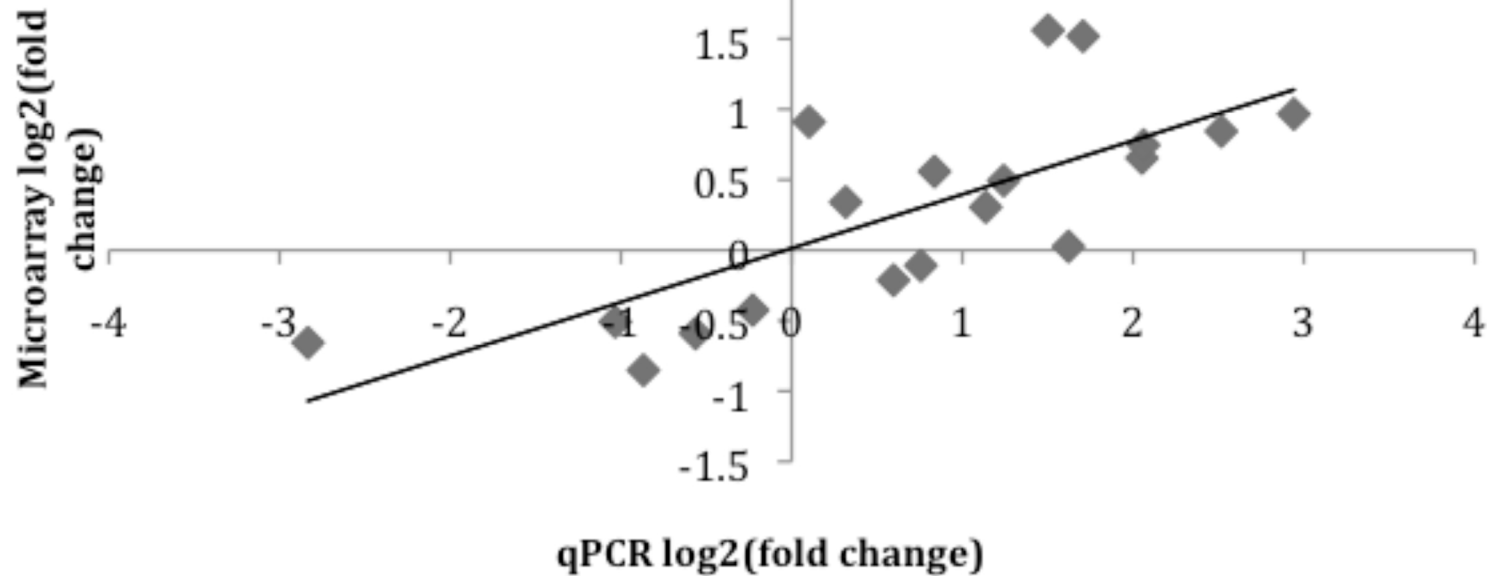

Supplement: Additional File 9 — Log2 Fold Change Correlations of selected genes detected by microarray and RTPCR. Log2 Fold change detected by RTPCR is depicted on the x axis, and microarray on the y-axis of 19 up- and down-regulated genes in the dataset. Spearman's rank correlation detected an R = 0.78 (p = 0.000075, DF = 17) correlation between microarray and qPCR detection of fold change. [file 1471-2164-12-449-S9.PDF]
